# Correlation between electroencephalographic markers in the healthy brain

**DOI:** 10.1038/s41598-023-33364-z

**Published:** 2023-04-18

**Authors:** Laura Päeske, Tuuli Uudeberg, Hiie Hinrikus, Jaanus Lass, Maie Bachmann

**Affiliations:** Department of Health Technologies, School of Information Technology, Tallinn University of Technology, 5 Ehitajate Rd, 19086 Tallinn, Estonia

**Keywords:** Biomedical engineering, Electrical and electronic engineering, Neurology

## Abstract

Mental disorders have an increasing tendency and represent the main burden of disease to society today. A wide variety of electroencephalographic (EEG) markers have been successfully used to assess different symptoms of mental disorders. Different EEG markers have demonstrated similar classification accuracy, raising a question of their independence. The current study is aimed to investigate the hypotheses that different EEG markers reveal partly the same EEG features reflecting brain functioning and therefore provide overlapping information. The assessment of the correlations between EEG signal frequency band power, dynamics, and functional connectivity markers demonstrates that a statistically significant correlation is evident in 37 of 66 (56%) comparisons performed between 12 markers of different natures. A significant correlation between the majority of the markers supports the similarity of information in the markers. The results of the performed study confirm the hypotheses that different EEG markers reflect partly the same features in brain functioning. Higuchi’s fractal dimension has demonstrated a significant correlation with the 82% of other markers and is suggested to reveal a wide spectrum of various brain disorders. This marker is preferable in the early detection of symptoms of mental disorders.

## Introduction

Mental disorders have an increasing tendency and represent the main burden of disease to society today. According to WHO's recent report^1^, nearly 15% of the world’s working population is estimated to experience a mental disorder. There is a high demand for effective methods and markers for the early detection and treatment monitoring of mental disorders.

Electroencephalography (EEG) is a method for the registration of brain electrical activity using scalp electrodes. The EEG signal is complex, containing information about physiological, emotional, cognitive, and other processes occurring simultaneously in a person. The EEG has proved to be an effective tool in neurophysiology used in clinical practice^2^. EEG markers describe the physiological state of the brain and can reflect the changes in brain electrical activity related to mental disorders. EEG markers can detect the objective symptoms of mental disorders and contribute significantly to the assessment of stress, depression, anxiety, and others. EEG is a non-invasive, patient-friendly, and easy-to-apply method that can be implemented in portable and wearable devices for regular personal use.

Mental disorders cause only mild alterations in EEG which are difficult to detect. Therefore, parallel to the traditional quantitative EEG based on the comparison of powers in different frequency bands of the EEG spectrum, different advanced methods have been developed for EEG analyses to detect mental disorders.

EEG signal is complex, stochastic, nonstationary, and nonlinear. This is the reason why the field of possible EEG markers used in the detection of mental disorders is so diverse. Different EEG markers can describe various features of the signal^3,4^. All EEG markers can be divided into three categories depending on the phenomena they describe: the traditional EEG frequency band power, the dynamic pattern of the signal in a single-channel EEG, or the brain functional connectivity in a multichannel EEG.

The changes caused by mental disorders have been detected by traditional EEG markers based on the powers of EEG frequency bands^5–8^. The resting state EEG alpha and beta powers increase in depression groups^5–7^. The EEG alpha power is suggested associated with depression severity^7^. In addition to increased band powers, the altered inter-hemispheric alpha power asymmetry^5,6^ and reduced coherence^5^ have been discovered in the same depression groups. The review of 184 studies has demonstrated that differences in EEG frequency bands powers are evident for many psychiatric disorders including depression, attention deficit-hyperactivity disorder, autism, addiction, bipolar disorder, anxiety, panic disorder, post-traumatic stress disorder, obsessive compulsive disorder and schizophrenia^8^. The power changes within specific frequency bands are not unique to one disorder but show overlap across disorders as well as variability within disorders^8^.

The various nonlinear and dynamic features of the signal in depression and other disorders can be described using more advanced EEG markers such as fractality, complexity, and frequency balance^9–15^. Detrended fluctuation analysis (DFA) shows higher values for depressed patients^9^ and also improves the diagnostic accuracy of Alzheimer's disease^10^. The Lempel–Ziv complexity (LZC) has indicated higher scores in both, schizophrenia and depression^11^. Higuchi's fractal dimension (HFD) has demonstrated good differentiation between the groups of depressive and healthy subjects^12–14^. The spectral asymmetry index (SASI) increases in the depressive group^14,15^ and is correlated with Hamilton Depressive Rating Scale for indoor patients^15^. The combination of nonlinear markers HFD, DFA, correlation dimension, and Lyapunov exponent markers provides a classification accuracy of depression of 90% which is higher than the classification accuracy for the linear EEG band powers markers 76.6%^13^. Different combinations of EEG linear (SASI, alpha power variability, relative gamma power) and nonlinear markers (HFD, DFA, LZC) have demonstrated rather close accuracies of classification for both, 0.88% for linear and 0.85% for nonlinear markers^14^.

The functionality of the brain, the coordination of neuronal activity in different brain areas, can be described by analyzing the connectivity between signals in different EEG channels^16–20^. Brain functional connectivity and EEG coherence increase in major depression^16–18^. The phase-sensitive markers, the imaginary part of coherence and synchronization, significantly contribute to the discrimination of depression^19,20^.

Despite reflecting various features in brain physiology, different EEG markers have indicated similar results in detecting mental disorders. EEG band power, Higuchi’s fractal dimension, Lempel–Ziv complexity, spectral asymmetry, and others have indicated quite a close accuracy in the evaluation of depression^13,14,21^. Based on these findings, two possible explanations can be proposed. First, the disorder causes different physiological changes reflected by the different features of the EEG signal and each marker detects a specific EEG feature. Second, the different EEG markers reveal the same EEG features and similar declinations in brain functioning.

Whereas the mild alterations in the EEG signal caused by mental disorders are hidden in the natural variability of the signal, the selection of appropriate markers revealing mental disorders is highly important. The selected EEG markers serve as the input data for classification algorithms. The classification accuracy depends strongly on the selection of the appropriate markers and not so much on the applied classification algorithms^22,23^. Therefore, the reasonable selection of EEG markers is especially important.

Only a few publications have been aimed to compare the effectiveness of different EEG markers^13,14,22^. The correlation between the EEG signals in different channels has been investigated^23,24^. To the best of our knowledge, the evaluation of the correlation between the markers and the independence between the information achieved from different markers has not been performed.

The current study is aimed to investigate the hypothesis that different EEG markers reveal partly the same EEG features and so provide overlapping information about the state of the brain.

To assess the hypothesis, the correlation between different EEG markers indicating various features of the signal is investigated. Some most frequently used EEG markers from band power, dynamics, and functional connectivity categories are selected for investigation, four from each category.

The band power markers describe the power of the signal inside the fixed EEG frequency bands and are not sensitive to the pattern of the signal. Theta band power (TBP), alpha band power (ABP), beta band power (BBP), and gamma band power (GBP) are selected for analyses in the first category.

The dynamics markers describe the pattern and the complexity of the signal. The four selected single-channel EEG dynamics markers describe various aspects of the complexity of the EEG signal. Higuchi’s fractal dimension (HFD) describes the self-similarity of the signal^25^. Detrended fluctuations analysis (DFA) describes the self-correlation of the signal and determines the self-affinity of the EEG signal^26^, while Lempel–Ziv complexity^27^ (LZC) describes the randomness of the signal. The spectral asymmetry index^12^ (SASI) describes the balance of low-frequency and high-frequency oscillations in the signal.

Functional connectivity markers describe the connectivity between different brain areas using multichannel data. Magnitude-squared coherence^28^ (MSC) describes the intensity of coherence between two signals. The imaginary part of coherency^29^ (ImC) characterizes phase relationships in the coherence between two complex signals^29^. Synchronization likelihood^30^ (SL) describes dynamical interdependencies between two signals. Mutual information^31^ (MI) describes the coherence of the information between two signals and can be considered a spatial analog of entropy.

The selection of markers considers linear (TBP, ABP, BBP, GBP, SASI, MSC, ImC) and nonlinear (HFD, DFA, LZC, SL, MI) EEG properties. The markers calculated in the time domain (HFD, DFA, LZC, SL, MI) and frequency domain (TBP, ABP, BBP, GBP, SASI, MSC, ImC) are included. The selection of functional connectivity markers is balanced between the phase-sensitive (ImC, SL) and phase-insensitive (MSC and MI) markers.

The study is planned in a way to minimize the impact of external factors and possible inter-subject variability due to the individual responses to a disorder on the EEG signals. The resting state eyes closed EEG of healthy people is analyzed in the study.

## Methods

### Subjects

The group of 80 volunteers, 38 (47.5%) female, and 42 (52.5%) male was selected for investigation. Their age varied from 19 to 75 years, with a mean age of 37 ± 15 years. They declared no mental or psychiatric disorders, epilepsy, brain injuries, or usage of narcotics or psychotropic medications. All the selected subjects were considered as healthy. The subjects were asked to abstain from alcohol for 24 h and from coffee two hours before the EEG recordings.

The study was conducted following the Declaration of Helsinki and was approved by the Tallinn Medical Research Ethics Committee. Before participating in the study, each subject signed informed consent.

### EEG recordings

The Neuroscan Synamps2 acquisition system (Compumedics, NC, United States) was used for EEG recordings. Electrodes were placed according to the extended international 10–20 system. The signals were recorded from 30 electrodes (Fp1, Fp2, F7, F3, Fz, F4, F8, FT7, FC3, FCz, FC4, FT8, T7, C3, Cz, C4, T8, TP7, CP3, CPz, CP4, TP8, P7, P3, Pz, P4, P8, O1, Oz, O2) using linked mastoids as reference. During recordings, eye movements were monitored using horizontal and vertical electrooculograms. Electrodes impedances were lower than 10 kΩ.

All EEG recordings were performed in the morning before noon. The resting state eyes closed EEG was recorded for 6 min. During recordings, the subjects were in lying positions in a shielded and dimly lit room. Earplugs were used to minimize external sounds.

The raw EEG was recorded in the frequency band 0.5–200 Hz at the sampling frequency of 1000 Hz.

### EEG preprocessing

The raw EEG signals were filtered into frequency band 1–45 Hz using a Butterworth filter.

To reduce the computing time, the signals were down-sampled to 200 Hz and recalculated to REST reference as preferable in EEG analyses^32,33^. The signals were divided into 20.48-s (4096 sample) segments. An experienced EEG specialist carefully inspected all segments and removed the segments with artifacts (ocular, muscular, or others). The first 10 artifact-free segments were used for further analysis. The signals were preprocessed using MATLAB (The Mathworks, Inc.).

### EEG analyses

#### Calculation of band power markers

First, the power spectral density (PSD) of the recorded EEG signal was calculated using the Welsh’s averaged periodogram method. The signal was divided into 50% overlapping sections and windowed by the Hanning window. Second, the markers were calculated as the mean of PSD over the frequencies within the fixed frequency bands TBP 4–7 Hz, ABP 8–12 Hz, BBP 13–30 Hz, and GBP 31–45 Hz.

#### Calculation of dynamics markers

The nonlinear dynamics markers (HFD, DFA, and LZC) were calculated in the time domain. Calculations were performed for ten 20.48-s segments. A nonlinear marker was determined as the mean value of the calculations’ results over ten segments. The HFD was calculated according to Higuchi’s original algorithm^25^ at kmax = 8^14,34^. DFA was calculated according to the published by Peng et al. algorithms^26^ applying the adaptation to EEG described by Bachmann et al.^14^. The calculation of LZC was performed based on the principles and algorithms published by Lempel and Ziv^27^ and Zhang et al.^29^ using the adjustment performed by Bachmann et al.^14^. SASI was calculated in the frequency domain summarizing PSD over the lower and higher EEG frequency bands and excluding the central alpha band from calculations^15^.

#### Calculation of functional connectivity markers

SL was calculated in the time domain following the detailed explanation of the method by the authors Stam and Van Dijk^30^, while the parameters were set as in Päeske et al.^34^, as such parameters ensure that the time–frequency characteristics of the signals are fully considered. MI was calculated using the algorithm derived by Frazer and Swinney^31^ following the method of the calculation for EEG signals published by Ibáñez-Molina and others^35^. MSC and ImC were calculated in the frequency domain, the algorithms were applied as described by Päeske et al.^36^.

The calculations of markers were done in MATLAB (The Mathworks, Inc.).

### Statistics

All EEG band power and dynamic markers were calculated for all EEG channels for each subject. All functional connectivity markers were calculated between 30 channels, in total 435 combinations were performed per marker for a subject. The averaged over all EEG channels values for a subject were used for statistical evaluation.

The null hypothesis for the difference between the values of markers was tested using the Wilkinson test. In total, (12 × 12–12)/2 = 66 comparisons between the pairs of 12 markers were performed on the same EEG database. The adjustment to multiple comparisons was done using Bonferroni correction. The corrected confidence level *p < *0.05/66 = 0.00076 was considered statistically significant.

The correlation between different EEG markers was assessed using the Spearman correlation coefficients. The null hypothesis for correlation coefficients was tested using t-test. The probability that the correlation between markers of two different categories is zero, decreases with the increase in the number of pairs n and the value of the correlation coefficient r. At the fixed number of pairs n = 80, the p score reaches the level of statistical significance *p < *0.00076 at the value of the correlation coefficient |r|> 0.37.

## Results

The nature of the EEG markers differs in different categories. Therefore, the results are presented separately in each of the markers’ categories followed by inter-categories correlations results.

### Band power markers

Wilkinson’s test indicated that the calculated values of different band power markers are mutually statistically significant (*p < *0.00076) in all combinations except TBP and BBP (*p = *0.03).

**Figure 1:**
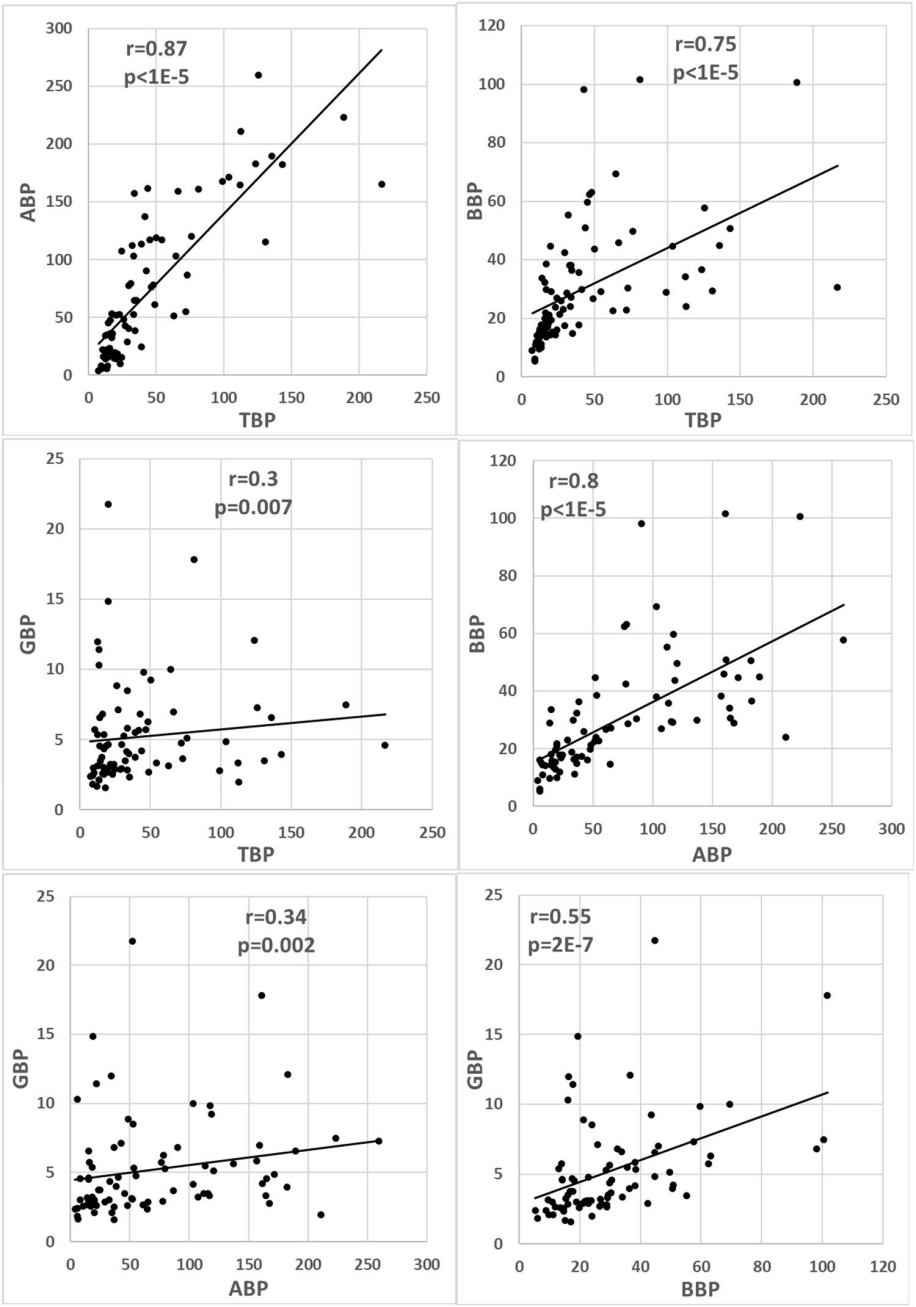
Correlation between various band power markers: TBP and ABP, TBP and BBP, TBP and GBP, ABP and BBP, ABP and GBP, BBP and GBP. The calculated Spearman correlation coefficients *r* between the markers and corresponding *p-*values are indicated (*n* = 80). The *p < *0.00076 (|*r*|> 0.37) indicates statistical significance.

The graphs in Fig. 1 present the correlations between the EEG band power markers. The calculated Spearman correlation coefficients and t-test *p-*values are indicated. The correlation is statistically significant between the markers of closer frequency bands TBP and ABP (*r* = 0.87), ABP and BBP (*r* = 0.80), whereas the correlation is somewhat less between TBP and BBP (r = 0.75) and insignificant between ABP and GBP (r = 0.34) as well as between TBP and GBP (*r* = 0.3). This finding may be related to the overlapping physiological processes in close frequency bands.

Four of six (66.7%) combinations between the band power markers indicate statistically significant correlations.

### Dynamics markers

Wilkinson’s test shows that the calculated values of all dynamics markers differ significantly in all combinations (*p < *0.00076).

**Figure 2:**
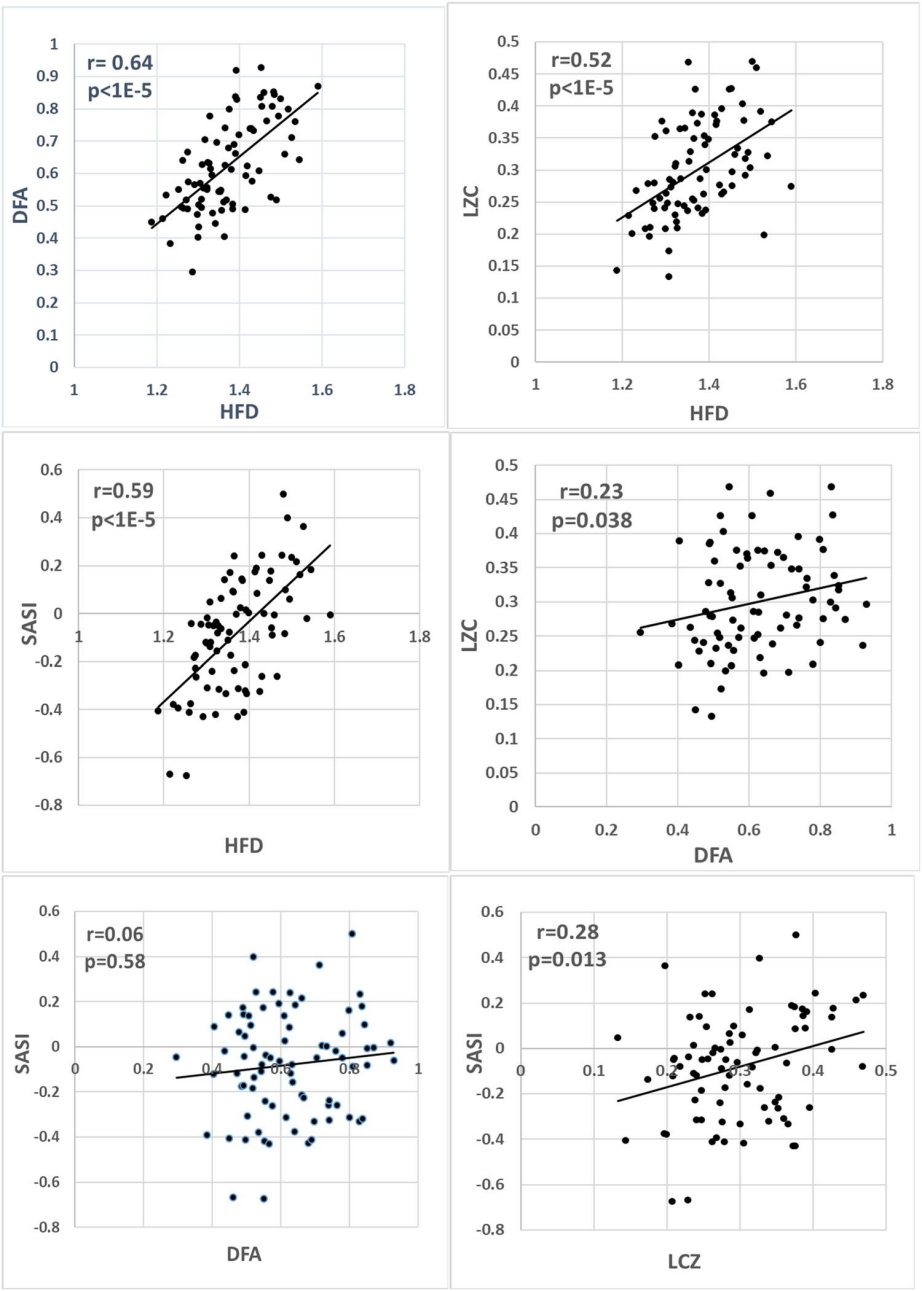
Correlation between various dynamics markers: HFD and DFA, HFD and LZC, HFD and SASI, DFA and LCZ, DFA and SASI, LZC and SASI. The calculated Spearman correlation coefficients *r* between the markers and corresponding *p-*values are indicated (*n* = 80). The *p < *0.00076 (|*r*|> 0.37) indicates statistical significance.

Figure 2 presents correlations between different dynamics markers. The calculated Spearman correlation coefficients and t-test *p-*values are indicated. HFD has a significant correlation with all other markers, maximal with DFA (r = 0.64), a little lower with SASI (r = 0.59), and with LZC (r = 0.52). The correlations between the other markers DFA, LZC, and SASI are not statistically significant. This finding supports the idea that HFD can incorporate partly the same EEG features as the DFA, LCZ, and SASI do. The other markers DFA, LZC, and SASI do not reveal mutually similar EEG features.

Three of six (50%) combinations between the dynamic markers indicate statistically significant correlations.

### Functional connectivity markers

Wilkinson’s test indicated that the calculated values of functional connectivity markers are statistically significant (*p < *0.00076) in all combinations except SL and MI (*p = *0. 297).

**Figure 3:**
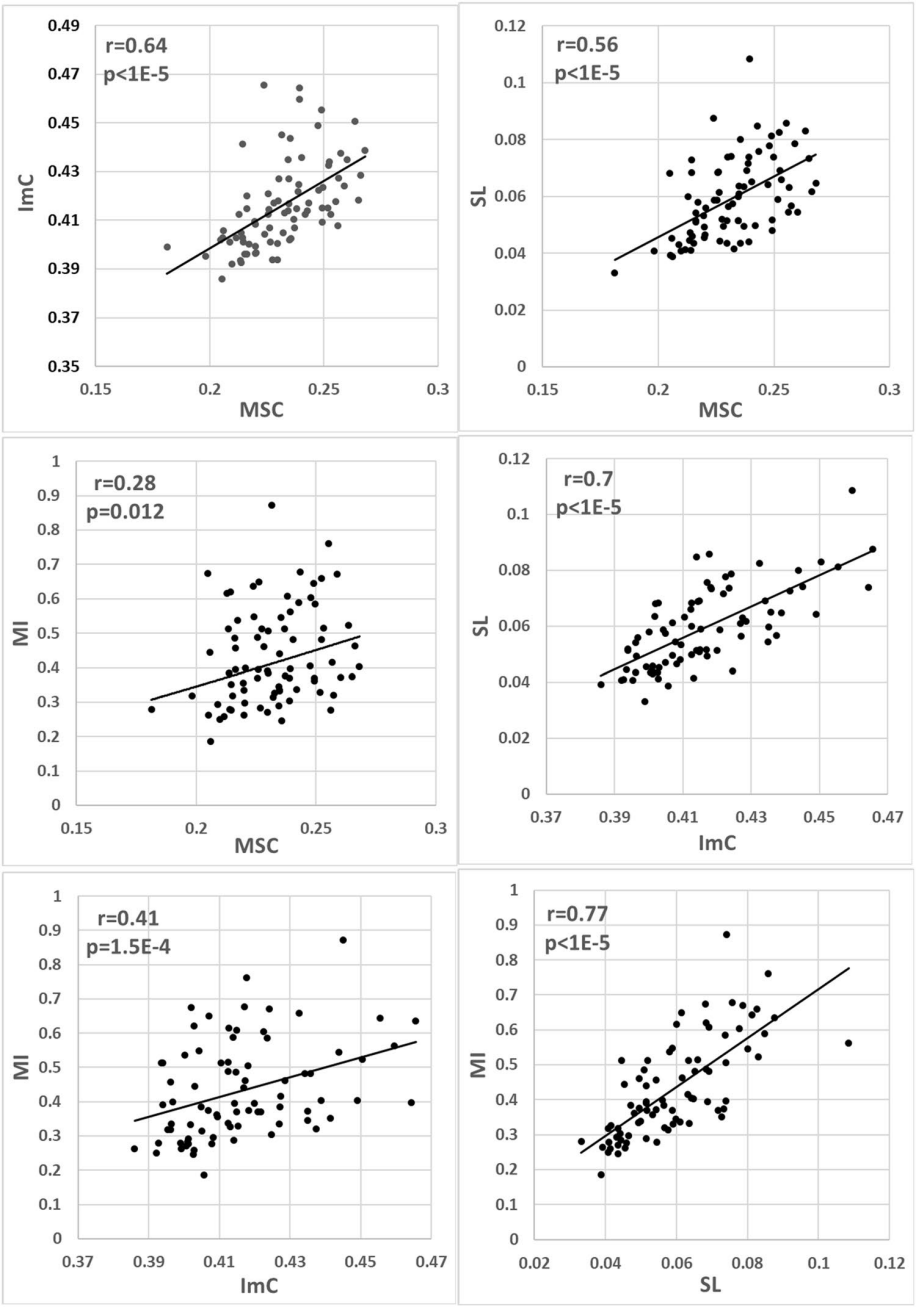
Correlation between various functional connectivity markers: MSC and ImC, MSC and SL, MSC and MI, ImC and SL, ImC and MI, SL and MI. The calculated Spearman correlation coefficients *r* between the markers and corresponding *p-*values are indicated (*n* = 80). The *p < *0.00076 (|*r*|> 0.37) indicates statistical significance.

Figure 3 presents the correlations between functional connectivity markers. The calculated Spearman correlation coefficients and t-test *p-*values are indicated. SL has a significant correlation with all other markers, the correlation coefficient between SL and MI is 0.77, between SL and ImC 0.7, and between SL and MSC 0.57. The expected correlation is between MSC and ImC (r = 0.64). Weaker but still significant is the correlation between ImC and MI (r = 0.41). This finding suggests that the various brain functional connectivity behaviors are mutually correlated and corresponding EEG features can be revealed by different markers.

Five of six (83.3%) combinations between the functional connectivity markers indicate a statistically significant correlation.

### Markers of different categories

Table 1 presents the calculated Spearman correlation coefficients between the EEG band power markers, dynamic markers, and functional connectivity markers and corresponding t-test *p-*values. The data in the table show that the correlation between the markers of different categories is not weaker than between the markers of the same category. The dynamic markers are negatively correlated with the band power markers (except GBP) and functional connectivity markers.

**Table 1:** The calculated Spearman correlation coefficients *r* between the pairs (*n* = 80) of different markers and corresponding *p-*values estimated by t-test. The *p < *0.00076 (|r|> 0.37) indicates statistical significance.

Marker	HFD	DFA	LZC	SASI	TBP	ABP	BBP	GBP	MSC	ImC	SL	MI
HFD												
* r*		0.64	0.52	0.59	− 0.77	− 0.85	− 0.52	0.11	− 0.35	− 0.42	− 0.68	− 0.85
* p*		0.00E+00	1.10E-06	1.18E-08	0.00E+00	0.00E+00	1.10E-06	3.20E-01	1.77E-03	1.17E-04	0.00E+00	0.00E+00
DFA												
* r*	0.64		0.23	0.06	− 0.72	-0.83	− 0.77	− 0.35	− 0.23	− 0.38	− 0.67	− 0.82
* p*	0.00E+00		3.81E-02	5.84E-01	0.00E+00	0.00E+00	0.00E+00	1.44E-03	4.42E-02	5.83E-04	0.00E+00	0.00E+00
LZC												
* r*	0.52	0.23		0.28	− 0.29	− 0.39	− 0.02	0.10	− 0.21	− 0.38	− 0.42	− 0.34
* p*	1.10E-06	3.81E-02		1.33E-02	9.11E-03	4.58E-04	8.93E-01	3.63E-01	5.96E-02	5.97E-04	1.42E-04	2.25E-03
SASI												
* r*	0.59	0.06	0.28		− 0.51	− 0.29	− 0.08	0.38	− 0.06	− 0.05	− 0.09	− 0.36
* p*	1.18E-08	5.84E-01	1.33E-02		2.33E-06	9.08E-03	5.05E-01	5.86E-04	6.00E-01	6.83E-01	4.05E-01	1.02E-03
TBP												
* r*	− 0.77	− 0.72	-0.29	-0.51		0.87	0.75	0.30	0.11	0.24	0.56	0.91
* p*	0.00E+00	0.00E+00	9.11E-03	2.33E-06		0.00E+00	0.00E+00	7.37E-03	3.24E-01	2.90E-02	1.21E-07	0.00E+00
ABP												
* r*	− 0.85	− 0.83	-0.39	-0.29	0.87		0.80	0.34	0.23	0.39	0.75	0.97
* p*	0.00E+00	0.00E+00	4.58E-04	9.08E-03	0.00E+00		0.00E+00	2.15E-03	3.85E-02	4.05E-04	0.00E+00	0.00E+00
BBP												
* r*	− 0.52	− 0.77	-0.02	-0.06	0.75	0.80		0.55	0.12	0.18	0.49	0.81
* p*	1.10E-06	0.00E+00	8.93E-01	5.05E-01	0.00E+00	0.00E+00		1.96E-07	2.90E-01	1.07E-01	4.74E-06	0.00E+00
GBP												
* r*	0.11	− 0.35	0.10	0.38	0.30	0.34	0.55		-0.25	-0.04	0.19	0.30
* p*	3.20E-01	1.44E-03	3.63E-01	5.86E-04	7.37E-03	2.15E-03	1.96E-07		2.42E-02	7.18E-01	9.82E-02	7.83E-03
MSC												
* r*	− 0.35	− 0.23	− 0.21	− 0.06	0.11	0.23	0.12	− 0.25		0.64	0.57	0.28
* p*	1.77E-03	4.42E-02	5.96E-02	6.00E-01	3.24E-01	3.85E-02	2.90E-01	2.42E-02		0.00E+00	8.13E-08	1.15E-02
ImC												
* r*	− 0.42	-0.38	− 0.38	− 0.05	0.24	0.39	0.18	− 0.04	0.64		0.70	0.41
* p*	1.17E-04	5.83E-04	5.97E-04	6.83E-01	2.90E-02	4.05E-04	1.07E-01	7.18E-01	0.00E+00		0.00E+00	1.50E-04
SL												
* r*	− 0.68	− 0.67	− 0.42	− 0.09	0.56	0.75	0.49	0.19	0.57	0.70		0.77
* p*	0.00E+00	0.00E+00	1.42E-04	4.05E-01	1.21E-07	0.00E+00	4.74E-06	9.82E-02	8.13E-08	0.00E+00		0.00E+00
MI												
* r*	− 0.85	− 0.82	-0.34	− 0.36	0.91	0.97	0.81	0.30	0.28	0.41	0.77	
* p*	0.00E+00	0.00E+00	2.25E-03	1.02E-03	0.00E+00	0.00E+00	0.00E+00	7.83E-03	1.15E-02	1.50E-04	0.00E+00	

The assessment of the correlations between EEG signal frequency band power, dynamics, and functional connectivity markers demonstrates that a statistically significant correlation is evident in 37 of 66 (56%) comparisons performed between 12 markers. HFD and SL are correlated with 9, MI and ABP with 8, TBP, BBP, and ImC with 7, DFA with 6, LZC and SASI with 4, and GBP and MSC only with 2 other markers. The level of correlation varies from 0.97 (between ABP and MI) to 0.38 (between LCZ and ImC, and GBP and SASI).

## Discussion

The results of the performed study support the hypotheses that different EEG markers reveal partly the same EEG features. The assessment of the correlations between band power, dynamics, and functional connectivity markers demonstrates that despite the values of the markers being statistically different, a statistically significant correlation is evident in 56%, (in 37 from 66) of the combinations between 12 markers.

Mental disorders can cause very different unpredictable alterations in the EEG signal varying in individuals. For early detection of mental disorders, a marker is required to be able to reveal a wide scale of possible symptoms. The ability of a marker to reveal disorders is based on both, the wide scale of EEG features incorporated by the markers determined by the number of correlated markers and the strengths of the correlations. A quantitative evaluation of different markers can be useful to compare their potential to reveal a wide scale of symptoms characteristic of various mental disorders. Therefore, an indicator describing the effectiveness of markers is used. The effectiveness of a marker *E*_i_ can be estimated as the product of the number of markers *N*_i_ correlated with marker *i* and the average value of the corresponding correlation coefficients *R*_i_.

**Figure 4:**
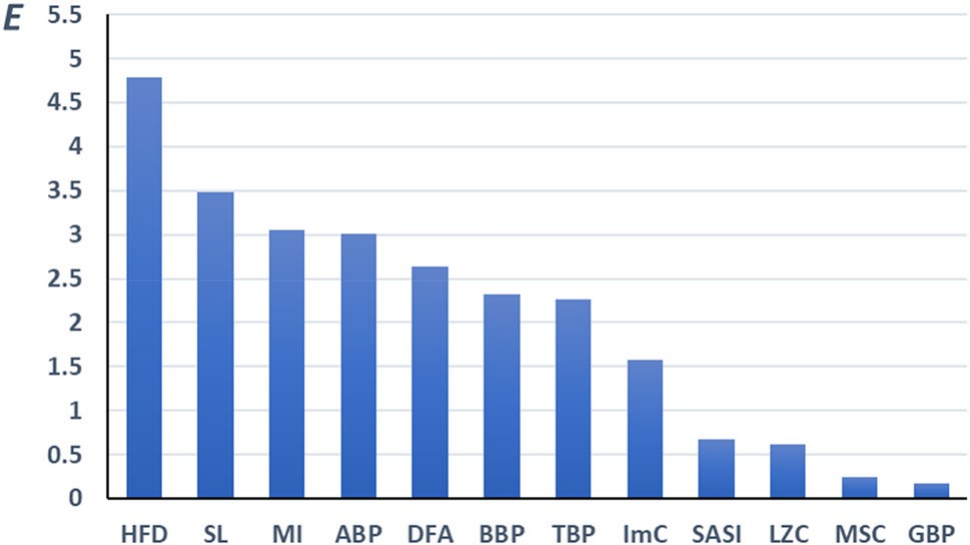
The effectiveness *E* of the EEG markers in detecting a wide spectrum of different EEG features. *E* = *NR*, where *N* is the number of markers correlated with the indicated marker and *R* is the average value of the corresponding correlation coefficients.

Figure 4 presents the effectiveness of each discussed in the current study markers. According to the graphs, the markers can be divided into three groups. The first group, HFD, SL, MI, and ABP, contains the markers expected to incorporate a wide scale of EEG features. The second group of markers DFA, TBP, BBP, and ImC covers a more specific part of EEG features. The markers GBP, MSC, LZC, and SASI from the third group can be useful for the detection of only a specific EEG feature. All the groups contain markers from all categories, band power, dynamics, and functional connectivity.

To provide a high-quality classification, the reasonable selection is an EEG marker from the first group correlated with many others and so incorporating very different features of the signal. The dynamic marker HFD is the marker of the highest effectiveness and is expected to incorporate a maximal part of the information from the EEG signal. This conclusion is supported by many studies where HFD has been successfully used for the detection of small alterations in EEG related to different factors such as depression, anxiety, or microwave radiation^22,37–40^. The traditional EEG band power marker ABP is the most commonly used band power marker which has shown good sensitivity in various applications^2,4,6,7^.

Two functional connectivity markers in the first group SL and MI demonstrate that both, phase relations and power are important in brain functional connectivity.

The marker from the second group DFA has demonstrated high classification accuracy for depression^13,14^. DFA combined with alpha band improved the classification accuracy of Alzheimer's disease^10^. The combination of ImC and cluster-span threshold has been reported optimal in graph theory analyses of depression^20^.

In addition, a second marker from the third group uncorrelated with the first one (e.g. GBP or LZC) can be useful, containing information about the features not incorporated in the first marker. This suggestion is supported by the analyses of depression EEG where the combinations of HFD and less correlated LZC lead to better classification accuracy compared to the combination of HFD and more correlated DFA^15^.

The effectiveness of GBP is low due to a very low level of gamma-band power in the EEG signal (less than 4% of total EEG power according to the scales in Fig. 1). Gamma-band power is not able to affect much the main features of the signal and the other markers. However, the information in GBP is independent of that in other markers and can add a noticeable contribution to the quality of classification in combination with other markers when used as an additional marker in classification^15^.

Table 1 shows that the correlation between the markers of different categories and different nature is not lower than the correlation between markers of the same category and similar nature. The correlation similar level of inter- and intra-categories correlations shows that the impact of the signal properties in the correlation between markers is not lower than the impact of the nature of the markers.

Today, no sufficient knowledge about brain functioning is available to explain the result of the study. Only some interesting trends in the relationships between the markers can be outlined.

There is a possibility that a high correlation of ABP with many other markers can be related to the higher power in the alpha band compared to other bands. The strength of the correlation of MI with band power markers follows the level of the power: 0.97 with ABP, 0.91 with TBP, 0.81 with BBP, and 0.30 with GBP. The strength of the correlation between SL and band powers shows the same trend. Such a trend agrees with the low effectiveness and correlation of GBP due to the low level of gamma band power. Despite that, gamma oscillations contain useful information and have been shown as a promising biomarker of depression^41^.

Interestingly, the correlation between the real and imaginal parts of coherence MSC and ImC 0.64 is lower than the correlation between two phase-sensitive markers ImC and SL 0.7. Such a trend supports the idea that the markers of similar signal property, phase, are more strongly correlated than the markers of different properties, phase and power. However, no specific general trends between linear or nonlinear markers calculated in frequency or time domain become evident.

The dynamic markers indicate a negative correlation with all band power markers (except GBP) and all functional connectivity markers. Fractal dimensions and other dynamic markers are scale-invariant and, in principle, independent of signal level. Their correlation with band power markers should be explained by processes other than dependence on the level of the signal. The decreasing of dynamics with an increase in connectivity is possible, but the mechanisms behind that are unknown.

The presented in Figs. 1, 2 and 3 and Table 1 results demonstrate that the two-channel functional connectivity markers are more strongly correlated than the single-channel band power or dynamics markers. The stronger correlation between the two-channel markers is a rather unforeseen result because the possible chaotic instabilities in two channels are stronger than in one. For example, the temporal stability of two-channel markers has been reported lower compared to single-channel markers^42^.

The results of the current study suggest that the HFD incorporating many various features of the signal is the best choice for EEG analysis to reveal signal features characteristic of early-stage mental disorders. The reported result may have a more general significance because the same markers can be used for signals other than EEG in several other applications.

The current study proves for the first time the correlation between different EEG markers. The difficulties in interpretation of the characteristic trends in the correlation between the markers underline the need for further investigations on the topic to get new knowledge about brain functioning and the relationship with EEG.

## Limitations of the study

There are several limitations in the study. The limitations are partly related to the concentration of the study on the evaluation of raised hypotheses,

The number of participants is limited due to the limitations in the volume of the study. The number is sufficient to provide the reliability of statistical evaluation for the whole. But this is insufficient for splitting subjects into smaller subgroups (male–female, old-young, etc.) because statistical comparisons become unreliable.

The results can be affected by factors other than the mental state of the brain. The possible impacts of gender and age are not considered. The possible dependencies of correlation on gender and age need further investigation.

The possible variations of the correlation in different brain areas and EEG channels are not discussed. Depression, and most likely other mental disorders, affect EEG signal in all brain areas^9,43^. Despite that, the correlation between markers can differ in different brain areas and channels. This problem needs future investigations.

Not all markers used by various researchers for the detection of symptoms of mental disorders have been discussed in the study. The selection of markers has been limited by the volume of the study. The interpretable makers describing different features of the brain activity used in more than one study have been preferred. Additional investigations on the correlation for the markers of interest can be performed in the future.

## Data Availability

Data are available upon request from the corresponding author.
